# Estimating Regions of Oceanographic Importance for Seabirds Using A-Spatial Data

**DOI:** 10.1371/journal.pone.0137241

**Published:** 2015-09-02

**Authors:** Grant Richard Woodrow Humphries

**Affiliations:** 1 Department of Neurobiology, Physiology and Behavior, University of California, Davis, Davis, California; 2 Department of Zoology, Center for Sustainability, Agriculture, Food, Energy and Environment, University of Otago, Dunedin, New Zealand; Institute of Ecology, GERMANY

## Abstract

Advances in GPS tracking technologies have allowed for rapid assessment of important oceanographic regions for seabirds. This allows us to understand seabird distributions, and the characteristics which determine the success of populations. In many cases, quality GPS tracking data may not be available; however, long term population monitoring data may exist. In this study, a method to infer important oceanographic regions for seabirds will be presented using breeding sooty shearwaters as a case study. This method combines a popular machine learning algorithm (generalized boosted regression modeling), geographic information systems, long-term ecological data and open access oceanographic datasets. Time series of chick size and harvest index data derived from a long term dataset of Maori ‘muttonbirder’ diaries were obtained and used as response variables in a gridded spatial model. It was found that areas of the sub-Antarctic water region best capture the variation in the chick size data. Oceanographic features including wind speed and charnock (a derived variable representing ocean surface roughness) came out as top predictor variables in these models. Previously collected GPS data demonstrates that these regions are used as “flyways” by sooty shearwaters during the breeding season. It is therefore likely that wind speeds in these flyways affect the ability of sooty shearwaters to provision for their chicks due to changes in flight dynamics. This approach was designed to utilize machine learning methodology but can also be implemented with other statistical algorithms. Furthermore, these methods can be applied to any long term time series of population data to identify important regions for a species of interest.

## Introduction

In the last two decades, technological advances have led to increased efficiency and lower costs of GPS units which allow scientists to track species for varying lengths of time in order to identify regions of importance [1–3]. These data are often used for predicting distributions which are used in conservation management. The deployment of GPS units comes with several downsides including significant financial cost, and detrimental effects on the animals being studied [4–6]. In many other cases, GPS data are sparse, covering only a limited temporal and spatial scale, with few tagged individuals [4]. In species where years of monitoring data may be available, it may be possible to overcome some of these downsides [7]. The sooty shearwater (*Puffinus griseus*) is a species of seabird that has a long term population dataset, with sparse GPS tracking data. In the Pacific, sooty shearwaters breed in New Zealand from October—April [8,9]. Sooty shearwaters are regarded as the most abundant bird in the Southern Ocean with a breeding population in the millions [10]. For many generations, chicks have been harvested by local Maori who have maintained personal diaries of their catch [11]. These diaries represent a long-term dataset of the numbers of chicks harvested per night for every year, and overall chick quality [12–14]. The harvest is split into two seasons, the nanao (April, when harvesters will pull chicks from burrows), and the rama (May, when harvesters collect nearly fledged chicks from the surface of the colony). Indices of both periods of the hunt and chick size were derived from these diaries [15].

The quantity of chicks available to be harvested could be determined by a number of interacting factors including the number and condition of adults returning to breed (and thus oceanographic conditions in non-breeding areas; [16]), and oceanographic conditions in the foraging regions during the breeding season [17]. The quality of chicks is most likely influenced by factors during the breeding season including the quantity and quality of prey items fed to chicks [18], and the duration of foraging trips by adults [19,20]; both of these measures can be impacted by physical ocean conditions [20,21]. It is therefore possible to determine the oceanographic regions that are important for these indices by examining specific oceanographic factors in a systematic fashion across a region.

Top marine predators like the Procellariiform seabirds are affected by physical ocean parameters because they rely on wind for dynamic soaring [20,22], and ocean processes to aggregate prey or increase prey availability [23]. Shaffer *et al*. [24] tracked 20 sooty shearwaters using geolocation archival (GLS) tags over two seasons and found that adults forage on long, offshore trips that lasted on average ~14 days in areas that are defined by strong upwelling and overlap with general patterns of myctophid distribution [22]. Short trips averages 2–4 days and were limited to coastal New Zealand waters. Because adult sooty shearwaters use relatively unchanging regions where they forage (core foraging areas), it is possible to quantify and test any oceanographic parameters which may affect harvest indices over time.

To this regard, it is possible to combine spatial techniques with the long-term datasets derived by Humphries [25] to infer potential regions of importance, which can be then ground-truthed using tracking data. Other studies have used a-spatial data to examine the potential distribution of seabirds [26–28], however all three methods involve examining distance to colony as either a ground-truthing device, or the primary factor for deriving distributional information. Either of these methods would be limiting for a pelagic seabird such as the sooty shearwater, which can travel thousands of kilometers from colonies while foraging during the breeding season.

This study aimed to test if a gridded spatial approach could be applied to a-spatial (population) data in order to identify regions of importance for a pelagic seabird during the breeding season. We also queried the models to examine potential mechanisms of behavior and distribution control. The methods presented in this study may be applied to any species for which long term ecological data exist, and blends long term ecological research with ecological niche modeling techniques.

## Materials and Methods

### Data

**Fig 1:**
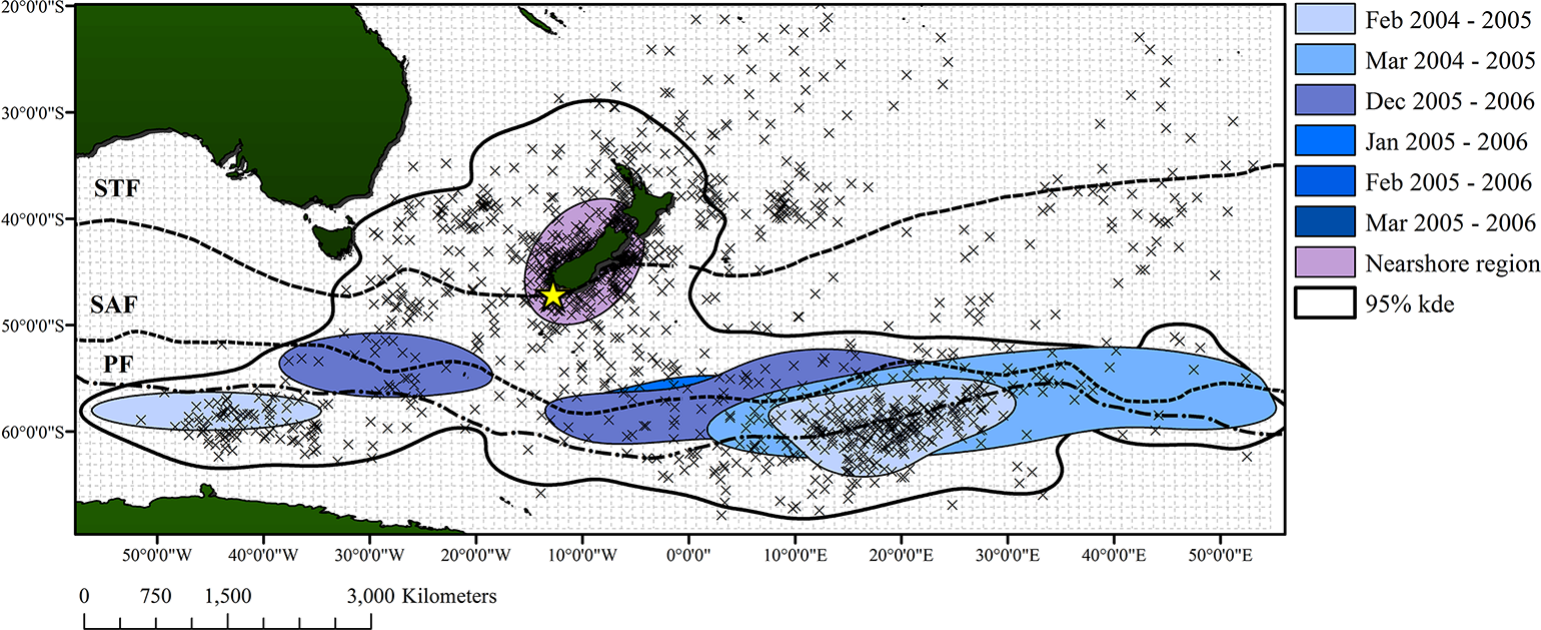
Map showing GLS data from Shaffer et al. (2006) for GLS birds tracked from Whenua Hou/Codfish Island (starred on the map) from January 2005 to March 2006. The 95% kernel density polygon for all data is represented by the largest polygon with a white background, while monthly 50% kernel densities for the offshore regions (offshore core foraging areas), and the 50% kernel density polygon for the nearshore region are represented by blue hues. The sub-Tropical front (STF), sub-Antarctic front (SAF), and Polar front (PF) are also represented on the map. The grid in the background represents the resolution of the environmental data used for modeling.

Archival geolocation (GLS) tag points were collected from 20 sooty shearwaters on Whenua Hou (Codfish Island), New Zealand, and Mana Island in 2004–2006 [24,29]. Each bird was captured from its burrow at night and fitted with a 6g GLS tag, representing <1.5% of the bird’s weight. Only one adult bird per burrow was fitted with a tag to limit the impacts on chicks[24]. However, for the purposes of this study, data were limited to birds tagged and recaptured on Codfish Island (*n* = 15), and filtered those points to represent only the breeding season (i.e., GLS points beginning on Nov 1st), and the approximate time which each individual bird left the colony to begin a Northward migration (approximately varies from March 31 to April 30). Of those birds, 7 were tracked through the 2004–2005 season and 8 through the 2005–2006 season. Offshore trips for birds were relatively consistent between years with most birds visiting the Southwest or Southeast foraging regions. One anomalous bird was removed from the analysis, because this bird left the colony early in the 2004–2005 breeding season and was likely a failed breeder. We therefore limited the spatial extent of our analysis to the extent of the GLS tracking data for the breeding season (Fig 1).

Kernel utilization polygons derived from the densities of GLS tracking points were calculated from GLS data for March 2005 and 2006 using the Kernel Density tool in ArcGIS 10.0 [30]. Kernel density analysis is used commonly to delineate important regions for birds with GLS tracking data [31–34]. Generally, regions where GLS tracking points are dense are assumed to be important for those individuals being tracked as it is where they spend the majority of their time, while regions with fewer GLS tracking points are considered ‘transit zones’[35]. That is, areas where birds could be located, but may not be foraging. The 95% density estimate was chosen in order to remove the effect of any outlying occurrences (i.e., points that occur away from the main aggregation that may occur and do not represent the majority of the population) and defined as the transit zone. The 50% kernel density polygon was also calculated and defined as the core foraging region [35].

Harvest index data were obtained from Humphries [25] for 1979 to 2010 to match the temporal resolution of the environmental data obtained. Harvest indices represented chick size, and mean tallies of birds harvested during the nanao (early) and rama (late) periods of the harvest. The harvest data were accessible due to a long-term partnership with the Rakiura Maori of New Zealand. The integration of science and traditional sources of knowledge are important as it builds trust between scientists and local committees, and allows for the creation of archived data which can be mined by future generations [36].

Open access environmental data were downloaded from the European Centre for Medium-Range Weather Forecasts (ECMWF) Interim analysis project (https://apps.ecmwf.int) at a spatial resolution of 0.75 x 0.75 degrees for the years 1979 to the present (all available years from the ERA interim analysis project). These data represent the output from numerical models of global climate used for weather forecasting. In general they are calculated via a series of formulae that relate variables to satellite derived global temperature patterns. It is possible that ECMWF variables are correlated because in some cases one product is a result of the relationship between another product and some constant value (e.g., wind speed derived from surface pressure). A list of the environmental data layers used in this analysis can be found in Table 1. Monthly climatologies of the variables were downloaded from 1979 to 2010 for use in oceanographic comparisons over a long time scale. Daily resolution climatologies were also downloaded from the ECMWF output to temporally match GLS data with the environmental variables. Data were processed in ArcGIS 10.0 (www.esri.com)[30], program R version 3.0.2 [37], and NCL version 5.1.2 [38]. Scale (i.e. temporal and spatial) of the data used can have vast effects on model inference and is not commonly addressed in spatial modeling studies [39]. I opted to use monthly resolution environmental data in this case because the harvest data used also represent monthly values. For example, rama indices are typically representative of April/May, while nanao indices are representative of March/April. I have dealt with spatial scale in this study by ensuring the resolution of all environmental data was identical. Sensitivity of these results to changing scale is not possible as long-term reanalysis projects such as ECMWF do not exist for the time period studied here.

**Table 1:** European Center for Medium Range Weather Forecasting (ECMWF; https://apps.ecmwf.int/datasets) data downloaded for use in modelling exercises.

Variable	Code	Units	Explanation
Charnock parameter	CHNK	-	Constant of atmospheric stress at ocean surface (Charnock 1955)
High cloud cover	HCC	%	Cloud cover at top level of ECMWF models
Low cloud cover	LCC	%	Cloud cover at lowest level of ECMWF models
Medium cloud cover	MCC	%	Cloud cover at mid-level of ECMWF models
Surface pressure	SP	Pa	Atmospheric pressure at surface of the ocean
Temperature at 2 meters depth	T2M	C	Ocean temperature at two meters depth
Total column water vapor	TCWV	kg*m^-2^	Vertically integrated total mass of water vapor
Sea surface temperature	SST	C	Temperature at top microlayer of ocean
Significant wave height	SWH	m	Combined wind wave and swell height
Sea surface temperature gradient	SSTG	%	Percent change of sea surface temperature
Wind speed	WSPD	m/s	Wind speed from 0 to 10 m above surface of the ocean
Wind direction	WDIR	-	Classified compass bearing of wind direction (16 classes)
Wind differential	WDIF	Deg	Difference between direction of travel and wind bearing

### Ethics statement

All protocols used by Shaffer et al. were approved by the Southland and Wellington Conservancies of the Department of Conservation, kai tiaki roopu, and the Institutional Animal Care and use Committees at UC Santa Cruz. Land access for Shaffer et al was granted by the Department of Conservation.

### Predictive analysis

Predictive analyses of data were performed using generalized boosted regression models (boosted regression trees/’gbm.step’; [40]) in R [37]. I opted for a machine learning algorithm because they are able to predict outcomes better than linear methods as they use the data to build predictions as opposed to forcing a model fit [41]. Also, they allow for the integration of many predictor variables in order to overcome biases when taking parsimonious (e.g., AIC) approaches. However, this method could be implemented using other statistical techniques such as generalized linear models or generalized additive models. Other implementations of generalized boosted regression modeling exist in Python, through the “scikit-learn” package [42], and Salford Systems predictive modeling suite [43], however R was chosen as it more easily integrates spatial data than Python, and is an open access platform (Salford systems predictive modeling suite is not, although a limited 30-day free trial can be downloaded). Moreover, scripts written in R can be easily shared and downloaded through web services such as “GitHub” making them more accessible to the general public, and also allow for smoother workflow.). Generalized boosted regression modelling is a machine learning algorithm that builds a series of regression trees, then minimizes error through cross validation tests. Due to the nature of cross validation and regression trees, models avoid over-fitting and thus allow for more flexibility in the selection of environmental variables to include in the model [40,44]. Model assessment in all cases was performed by way of cross-validation. Although machine learning techniques offer powerful predictive output [41], relationships between the response variable and explanatory variables are often difficult to interpret. In order to alleviate this, the response variables were plotted against significant explanatory variables and examined in linear space for basic interpretation as mechanistic relationships were not the primary goal of this study.

### Spatial model of harvest indices

The conceptual framework behind this method is derived from commonly used presence/absence spatial modeling techniques. In these models, the relationships between species occurrences and environmental variables are extrapolated to a regular grid in order to determine the probability of an organism occurring within a grid cell. That is, the ecological niche of the organism is quantified and then projected on a map. In the case of this study, occurrence data is being replaced with population index data, extrapolating the relationship to grid cells within the study region (as defined in Fig 1), and then examining where the “best” models (i.e., pixels with highest assessment values) occur. In other words, we are capturing the ecological niche of the population data and then projecting it in space.

Monthly mean values of the environmental variables for March from 1979 to 2010 were used in order to represent the feeding period which would most influence chick size during the harvest (this is because adults have been reported to leave in early April; [8]). Personal observations from the 2013 breeding season also suggest that March is important in chick growth and may represent a threshold month by which birds are forced to either abandon or continue feeding chicks.

Generalized boosted regression models were run with the same settings for every 0.75° x 0.75° grid cell, and for each cell, root mean squared error of the model (calculated by leave-one-out cross validation) was mapped. Although only few data (*n =* 31 years) and 12 predictors, leave-one-out cross-validation was also used to measure error loss across the boosting process to ensure over-fitting was not occurring. These results were then mapped in relation to frontal regions, which were found to be important foraging zones for sooty shearwaters [24].

### Comparing indices to oceanographic variables

GLS data from March 2005 and 2006 were used to compute both the nearshore and offshore core foraging areas for March (50% kernel density polygons), corresponding to the time when chicks are at their peak weight. Nearshore and offshore areas were calculated because of a hypothesized dual foraging strategy that may be implemented by sooty shearwaters during the breeding season [45]. Spearman correlation tests were performed to measure the correlation and significance between each of the mean oceanographic parameters for the core foraging areas, as well as for any other regions highlighted as important by spatial models. Spearman correlations were used to calculate correlation coefficients for non-linear relationships in the data with a bonferroni corrected *p*-value for repeated hypothesis testing. The bonferroni corrected *p-value* is a conservative measure used to limit the number of significant statistical relationships that might be noted simply due to chance when testing relationships between one response and multiple explanatory variables. In machine learning methods, *p*-values are typically not used for mechanistic inference [41], but we use them here to highlight particularly strong relationships between the response and predictor variables.

## Results

### Spatial modeling of harvest indices

**Fig 2:**
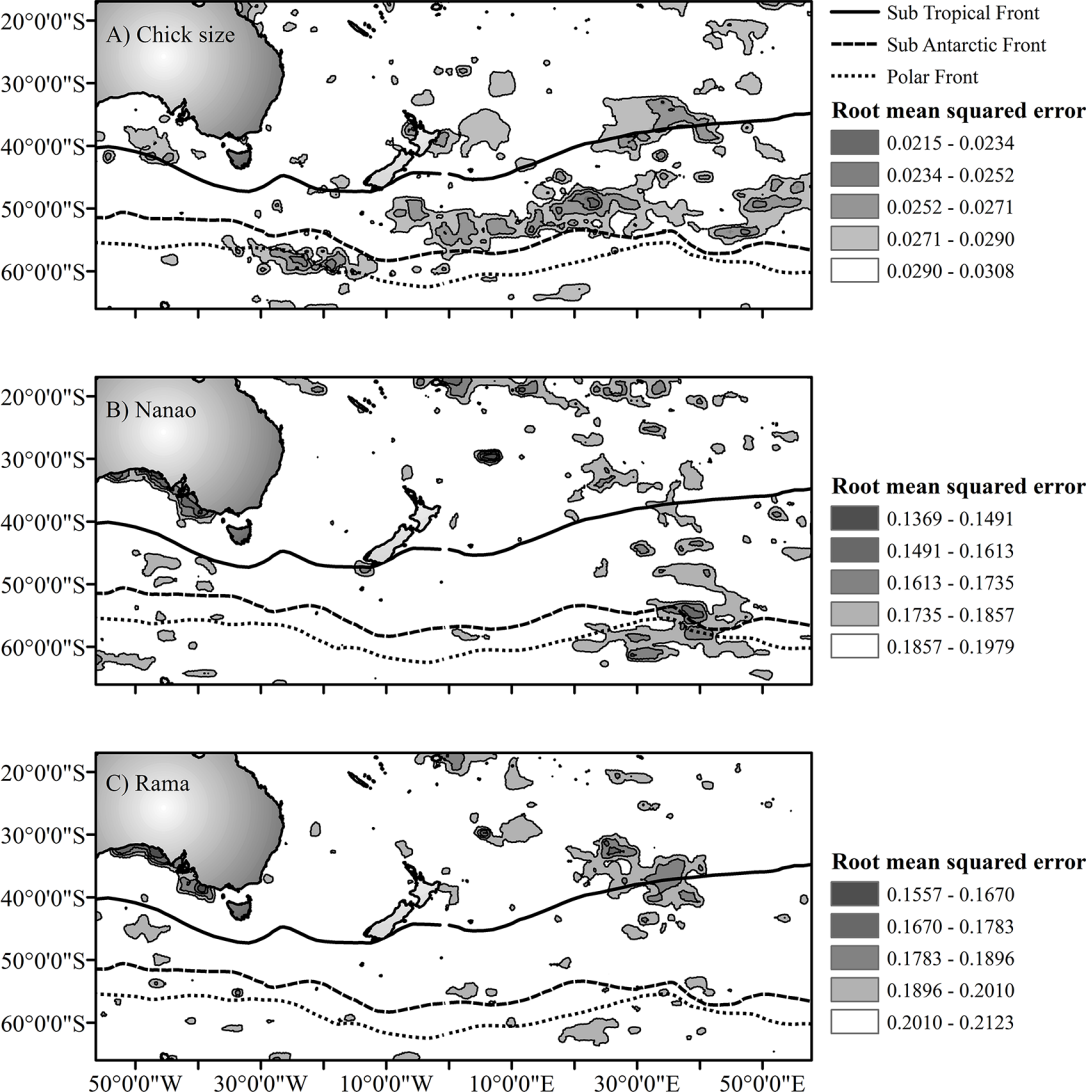
Mapped root mean squared error for generalized boosted regression models in the study area. Areas with the lowest root mean squared error represent regions where oceanographic factors for the month of March from 1979–2010 best capture the variability in the chicksize (a), nanao (b), and rama (c) indices from Humphries [25]. Frontal regions are depicted to demonstrate the boundaries of Southern Ocean zones.

Maps of the root mean squared error of the chick size, nanao and rama indices (Fig 2A, 2B and 2C respectively) show the areas where oceanographic conditions in March best explain variation in the datasets. In all three cases, maps are moderately patchy, with areas of low root mean squared error occurring to the North east of New Zealand and even off the South coast of Australia. The map of chick size model assessments has the most pronounced patterns, showing two regions to the Southwest and Southeast of New Zealand with root mean squared errors of 0.0215–0.0234. The region to the Southeast falls in the sub-Antarctic water area between the sub-Tropical and sub-Antarctic fronts, while the Southwestern region falls directly on the Polar front. The latter shows some overlap with the 50% kernel densities in the Southwestern areas (Fig 1). There is also a region around the North Island of New Zealand, and two regions to the far-east and to the northeast (above the sub-Tropical front) with root mean squared errors between 0.0234 and 0.0252. The nanao patterns are best explained in the eastern area of the sub-Antarctic front and Polar front regions and directly off the South coast of Australia with root mean squared errors for 0.1369–0.1613. Patchy areas of low root mean squared error are found in the Northern parts of the study area with values from 0.1491–0.1735. Of interest for the nanao is a small area directly around Stewart Island, New Zealand with root mean squared error of 0.1491–0.1613, where all of the colonies used to calculate the indices are found. Patterns in the rama are best explained in two regions; South of the Australia coast and east of New Zealand along the sub-Tropical front with root mean squared errors between 0.1557 and 0.1783.

Because the most pronounced patterns are found in Fig 2A (for chick size), most of the analysis focuses on this feature. Also, due to the fact that most of the birds from the GLS data travelled to the Southeast region [24] and because these areas are regions that are easily reached by sooty shearwaters during breeding seasons foraging trips, further focus was placed on the region of the sub-Antarctic water where the chick size indices are best explained.

### Oceanographic relationships with harvest indices

**Fig 3:**
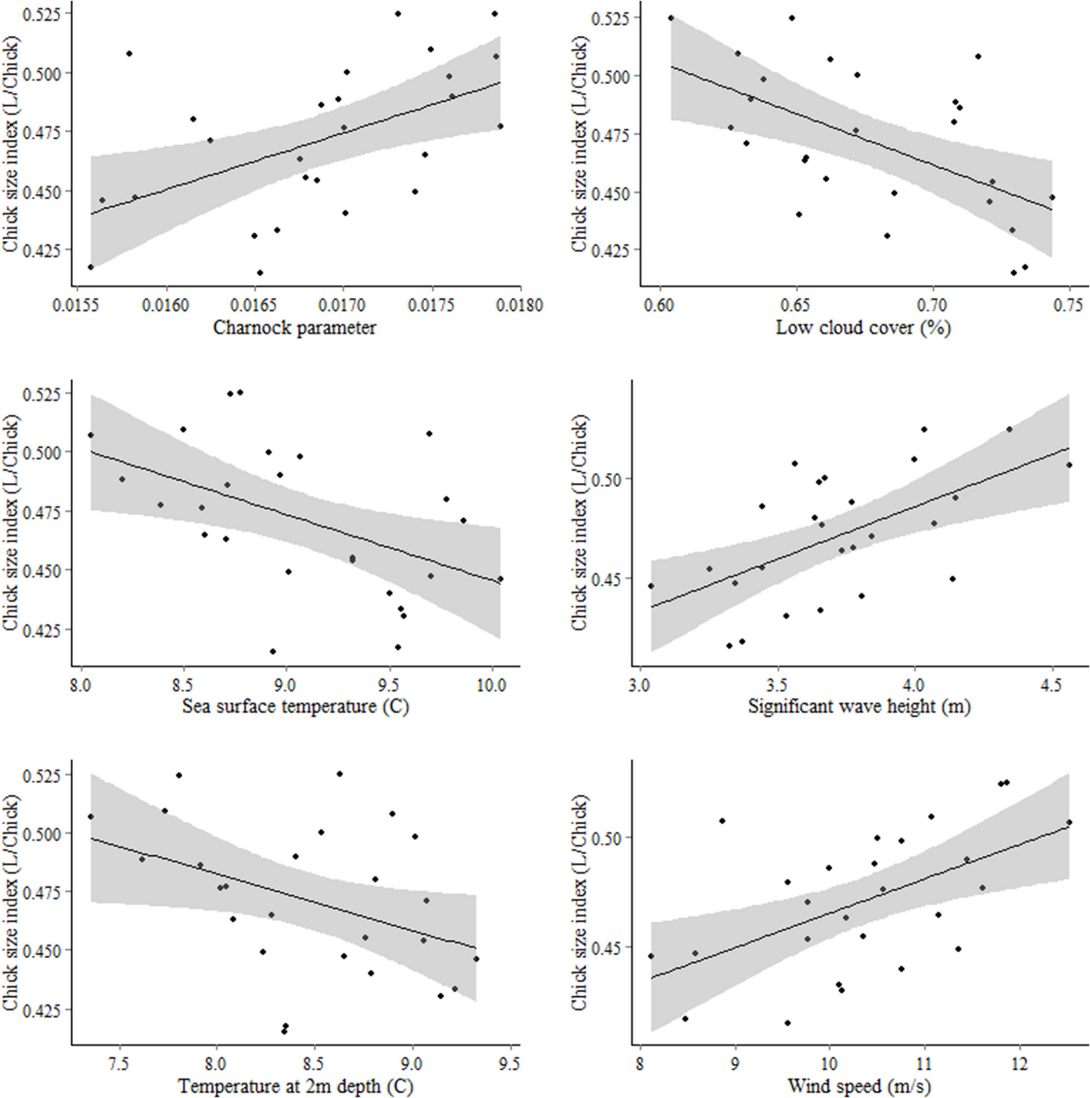
Linear relationships with oceanographic variables significantly correlated with the chick size index in the sub-Antarctic water region as per Table 2.

Spearman correlations for the chick size index in the sub-Antarctic water region show significant positive relationships with charnock parameter, significant wave height and wind speed (correlation coefficients of 0.57, 0.56 and 0.55 respectively). When values of atmospheric stress (charnock) in the sub-Antarctic region are high (between 0.0175 and 0.018; due to higher wind speeds, strong currents and high waves), mean chick size index values are between 0.475 and 0.525. These high chick size index values are also associated with mean wave height > 4.0m and wind speeds > 11 m/s. Lower chick size indices between 0.425 and 0.45 are associated with charnock values between 0.0155 and 0.016, wind speeds < 9m/s and waves <3.5m in height in the sub-Antarctic region. These relationships are presented in sample partial dependence plots in S1 Fig to demonstrate some of the output possible when using generalized boosted regression models. By contrast, chick size shows a significant negative relationship with low cloud cover, with lower chick size indices (<0.45) being associated with >73% low cloud cover (Table 2; Fig 3; correlation coefficient of -0.57). A significant negative correlation also existed between chick size index in the southeast core foraging region and total column water vapor (correlation coefficient of -0.51). In the sub-Antarctic water region, the nanao index had a negative significant correlation to sea surface temperature (-0.53), while in the core foraging region it had a negative significant correlation to significant wave height (-0.51; Table 2). However, it is important to note here that evidence to support the nanao harvest index was low due to lack of strong correlations between diaries therefore it is possible these relationships are spurious. No significant relationships were found between the rama and any of the oceanographic features in the three areas of interest, nor were there any significant relationships in the New Zealand coastal waters.

**Table 2:** Spearman correlations for March mean values of oceanographic variables from 1979–2010 versus three harvest indices within each of the identified oceanographic regions that are important for sooty shearwaters. Negative directionality in a relationship is shown by a minus sign in front of the correlation coefficient.

	sub-Antarctic water			Core Foraging Area			New Zealand coastal		
Variable	chicksize	rama	nanao	chicksize	Rama	nanao	chicksize	rama	nanao
Charnock parameter	0.57*	0.32	0.41	0.37	0.17	0.27	0.4	-0.04	-0.03
High cloud cover	0.27	-0.04	0.13	0.14	0.09	-0.08	0.09	-0.1	0.19
Low cloud cover	-0.57*	-0.32	-0.18	-0.36	-0.24	-0.05	-0.24	0.22	0.13
Medium cloud cover	0.25	0.1	0.12	0.28	0.3	0.15	0.07	0.1	-0.11
Surface pressure	-0.23	-0.13	-0.17	-0.4	-0.32	-0.28	-0.08	-0.11	0.01
Sea surface temperature	-0.47	-0.24	-0.53*	-0.31	-0.28	-0.48	-0.1	0.22	-0.05
Sea surface temperature gradient	-0.26	0.03	0.15	-0.42	-0.3	-0.37	0.39	0.11	0.34
Significant wave height	0.56*	0.22	0.37	-0.31	-0.27	-0.51*	0.34	-0.03	-0.04
Temperature at 2m depth	-0.42	-0.2	-0.46	0.35	0.19	0.27	-0.01	0.17	0.01
Total column water vapour	-0.19	-0.17	-0.24	-0.51*	-0.15	-0.45	0.11	0.23	-0.01
Wind speed	0.55*	0.29	0.37	0.26	0.2	0.2	0.34	-0.03	-0.04

* Spearman correlation is significant with bonferroni corrected *p* < 0.0045

## Discussion

Typically, GPS tracking data are used to examine where top predators forage during the breeding season. This is a straight-forward and direct method of obtaining important information about distribution. However, in many cases, GPS data may not be readily accessible, and there are many implications on the impacts of using tagging devices on animals [4–6]. This study tested the use of a-spatial data as a way of inferring important oceanographic regions or conditions for seabirds which could help limit the use of invasive GPS tags while promoting long-term ecological research. With long-term datasets, these methods can be applied in order to supplement information on seabird distribution when tracking data are not available, and build baseline population data for long-term monitoring of ocean health.

### Spatial models of harvest indices

The spatial models of the harvest indices show patches across the entire study area where the suite of environmental factors used best capture variation in the harvest indices. For many of these regions, it is likely that the relationship with the indices is due to correlation, and oceanographic conditions do not directly influence the indices from a mechanistic perspective. For example, according to GLS tracking data, patches off the southern coast of Australia with low RMSE values do not correspond to areas where sooty shearwaters visit on foraging trips during the breeding season. Another large patch of low RMSE values to the far east of the sub-Antarctic water region has little overlap with GLS data, save for a few locations to the southwest of the patch along the sub-Antarctic front. The patch that lies along the sub-Tropical front in the East of the study region shows some overlap with GLS locations, however these are locations from birds that were departing the breeding islands on the migration northwards, so this is not likely an area that would affect chick quality. A small patch around the North Island of New Zealand could be plausibly visited by birds from Whenua Hou (Codfish Island), however the majority of the GLS data suggest these birds tend to stay more around the South Island. The only region that overlaps well with the GLS data is the patch to the Southwest, which lies along the Polar front, similar to where adults foraged during the 2004/2005 and 2005/2006 breeding seasons. The large patch of low RMSE values to the Southeast of New Zealand is also of interest to us because this is a region that birds must pass through in order to arrive at the Southeast foraging area according to the Shaffer et al. (2006) data. If conditions in this region do not facilitate the travel of birds from the colony to the foraging site, then birds will invariably take longer on full trips, which would have detrimental effects on quality of chicks.

The regions which best describe the variation in the nanao harvest index (Fig 2B), but do not coincide with the distribution of sooty shearwater adults (based on the GLS data) are along the Southern Australian coast, and patches to the North of the study area in Sub-Tropical waters. One area of note is the patch of water immediately surrounding Stewart Island, which has low RMSE values for the nanao however, GLS data seem to suggest birds forage more frequently off of the South Island, and in the Southeast core foraging regions, at least for the 2004/2005 and 2005/2006 breeding seasons [24]. The patch of low RMSE values to the Southeast region however overlaps with the Southeast core foraging area based on the GLS data. The suite of oceanographic factors in this case could represent potential factors that influence the types of prey birds are bringing back to their young. For example, sea surface temperature shifts in this region may indicate a change in the strength or position of the Polar or sub-Antarctic fronts, which would have effects on how certain prey items would be distributed within a region [46]. Lower quality food in the adult foraging regions could lead to longer periods of time at sea [45], or reduced quality of food returned to the young, which would lead to increased chick mortality.

There were few regions which best explained the variation in the rama index data. Some patches of low RMSE were noted in the Northern parts of the study region, and another area off the South coast of Australia, which overlapped with the same region for the nanao index. The most obvious patch for the rama was the patch east of New Zealand along the sub-Tropical front, which overlaps heavily with good model results from the chick size index. This area overlaps with GLS data from birds that were heading North at the end of the 2004/2005 breeding season.

### Oceanographic drivers of the harvest indices

Based on results from the spatial models, the sub-Antarctic water region south east of New Zealand was included in the investigation into the oceanographic controllers of the harvest indices. For the chick size index, there was a significant negative relationship with low cloud cover that may be due to random correlation as it could be possible that increased low cloud cover might be indicative of lower wind speeds and does not have any direct consequences on chick quality. Significant positive correlations were found with variables that may be associated with how a bird forages at sea (i.e., wind speed, charnock, and wave height). Increased wind speed or atmospheric stress (i.e., high values of the charnock parameter) may allow birds to fly faster and more efficiently through the sub-Antarctic water region, which would allow adults to reach foraging areas faster and return to the colony to feed chicks, thus improving chick quality over the course of a season. Humphries [25] found that factors which represented turbulence (wind speed, wave height, etc…) in the sub-Antarctic water region influence total trip duration of sooty shearwaters. In more turbulent conditions, birds were able to take shorter trips, which would directly influence chick quality. It has also been shown in other studies that procellariiform seabirds are highly dependent on winds for flight [20,22,47].

Within the core foraging area, a negative relationships existed with total column water vapour. A negative relationship with total column water vapour may be related to the relationship with the sea surface temperature in the sub-Antarctic water due to increased temperatures causing more evaporation. Increased atmospheric water vapour causes cloud formation [48], which would prevent light from reaching the surface of the ocean and could slow productivity [49]. However, a lagged effect would be expected and therefore the relationship may simply be a non-causative correlation.

Relationships for both the nanao and rama indices were generally less pronounced in all regions, with the rama indices showing no significant linear correlations with any oceanographic variable. There could be two reasons for this: 1) the oceanographic variables that would affect the rama index are not found within the study area. For example, the numbers of birds available to be harvested may be more affected by conditions in the wintering grounds. 2) Only conditions for March were examined and because it is likely that certain indices may be affected by conditions from November–February, patterns were undetectable.

The nanao index shows a negative correlation with sea surface temperature in the sub-Antarctic water region, and with significant wave height in the core foraging area. The negative relationship with significant wave height is of note because it may be opposite to conventional thinking, and opposite to the relationship in the sub-Antarctic water region. Humphries [25] did not find any behavioral relationship between significant wave height in the core foraging area and total time at sea, and it could therefore be possible that this is a non-causative relationship as an increase in significant wave height would be expected to increase a bird’s ability to forage because it indicates more turbulent and windy conditions, which would facilitate flight [47,50] and olfactory search [51–53]. However, Humphries *et al*. [15] reported that the nanao index may not be a suitable scale to use due to the lack of correlation between diaries, and many of these correlations described here may be due to statistical noise.

It is important to note that many of these correlations are low to moderate, with a maximum r value of 0.57. There could be several things occurring here: 1) The role of only 11 physical oceanographic parameters were examined, and there is the possibility that there are other, unknown physical factors that have not been included in these models. 2) Biological components of the ecosystem (e.g., primary productivity or zooplankton distribution) have not been included here, but have been linked to the distribution of sooty shearwaters [29]. 3) Only physical parameters for March were examined. It is very likely that parameters like the nanao and rama indices are highly influenced by variables from November to March because they would represent the cumulative effects of oceanographic systems over the course of the breeding season. It would be reasonable to assume that the chick size (which is measured in April and May during the harvest) would be most affected by conditions during peak chick size in March, which may explain the generally stronger results obtained for this index. 4) We have selected a spatial extent which is limited to the GLS data used, while sooty shearwaters migrate to Japan, Alaska and California during the non-breeding season[24]. Because conditions in these regions may affect adult survival (and thus have impacts on the nanao and rama harvest indieces), we may be omitting important details for the population indices themselves. However, many of these points do not detract from the method presented in this study, which has identified a region of importance (sub Antarctic water) in determining size of chicks, based on a-spatial data.

### Data quality and quantity issues

There are several caveats to the data used that must be discussed prior to making conclusions on potential oceanographic drivers. Firstly, GLS data obtained from Shaffer et al. [24] only represent a very small subset of birds (*n* = 14) for part of the 2004/2005 and 2005/2006 breeding seasons. Although this represented 14 different birds tracked over two seasons, Small sample sizes like this could limit the statistical integrity of any conclusions [2,4], particularly for a species like the sooty shearwater due to its large population size. No more data were available for these birds so the results must be considered in this regard. Secondly, GLS data were from one breeding colony (Whenua Hou/Codfish Island). This island was not represented in the harvest indices used for this analysis however, studies comparing Whenua Hou (Codfish Island) to the harvesting islands via burrow counts show that population trends are comparable [12]. Thirdly, oceanographic data used were obtained from model output as opposed to primary sources (i.e., satellite or direct measurements). Thus, there is a risk of correlation between variables. However, due to the nature of generalized boosted regression models, it is reasonable to use correlated variables and still obtain meaningful predictions. This is because decision tree splits are determined based on the variables which lower the overall variance in the response data. When two variables are highly correlated, either one of those variables may be selected at random to explain the variation in the data. As the generalized boosted regression algorithm iteratively builds more trees, either of the correlated variables may be selected at each step, which separates the effect of the correlation. Testing of model performance occurs iteratively using cross-validation to ensure no over-learning is occurring at each step. Adding correlated variables into a generalized boosted regression model is therefore justifiable when the end result is predictions. The issue becomes conflated when attempting to disentangle mechanistic relationships, which is a goal of many ecologists. A traditional way to alleviate problems that may arise is to predict to independent datasets and trim explanatory variables from the models until the best combination of factors is determined. Another complicated but potentially more powerful approach would be to focus on predictive accuracy, which may involve the inclusion of large numbers of predictor variables. In this case, interpretation of mechanisms take into account many variables and may be more representative of reality [41]. In this case I was limited by the amount of data available, therefore an exploration into important relationships is made in a more targeted fashion using simple linear regression.

## Conclusions

The method presented in this study can be applied to any study system where long-term monitoring data exist in combination with maps of environmental data representative of the same time span. This type of systematic approach could aid in delineating regions of importance for species that are either difficult to track (e.g., small shorebirds), or lacking in tracking data. Similarly, this approach could re-inforce any conclusions that are made using only tracking data, and help to understand driving mechanisms in species distributions. The amount of tracking and long term monitoring data has been increasing steadily, and this method can increase our ability to predict important regions for a wide range of species, while limiting over-use of potentially detrimental tracking technologies.

## Supporting Information

S1 Fig Partial dependence plots of wind speed and significant wave height depicting relationships between both variables and the partial dependence values of chick size. When partial dependence values are higher, there is a more positive relationship towards higher predicted values.
(TIF)
