# Supplementary figures and images for: Estimating Regions of Oceanographic Importance for Seabirds Using A-Spatial Data
Source: PLoS One. 2015 Sep 2;10(9):e0137241. doi: 10.1371/journal.pone.0137241 (PMC4557983; doi:10.1371/journal.pone.0137241)

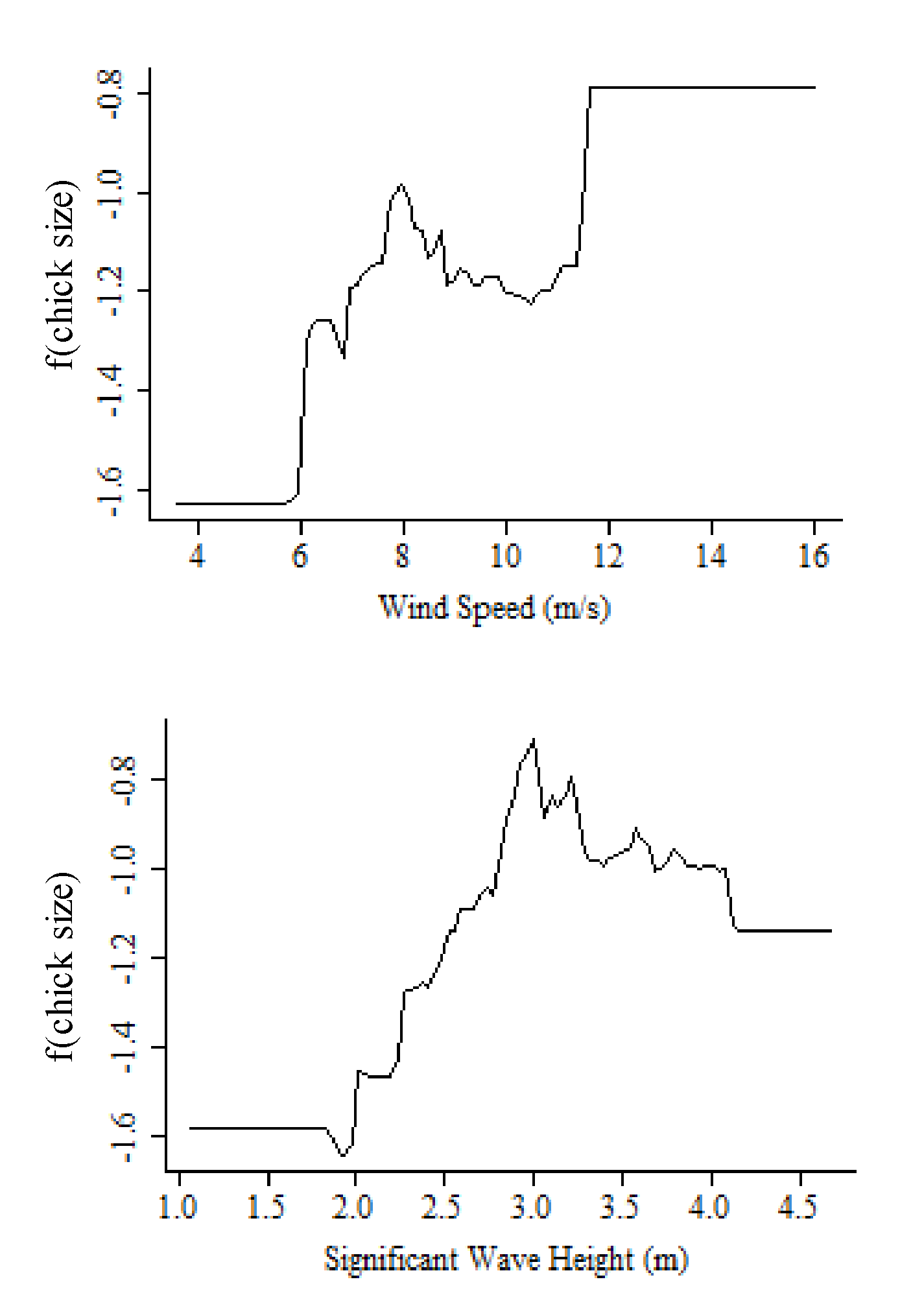

Supplement: S1 Fig — When partial dependence values are higher, there is a more positive relationship towards higher predicted values. (TIF) [file pone.0137241.s001.tif]
